# Structural and temporal patterns of the first global trading market

**DOI:** 10.1098/rsos.180577

**Published:** 2018-08-22

**Authors:** Ana Sofia Ribeiro, Flávio L. Pinheiro, Francisco C. Santos, Amélia Polónia, Jorge M. Pacheco

**Affiliations:** 1 CIDEHUS, Universidade de Évora, Apartado 94, 7000-809 Évora, Portugal; 2 CITCEM, Faculdade de Letras, Universidade do Porto, 4150-546 Porto, Portugal; 3 Departmento de História e Estudos Políticos Internacionais, Universidade do Porto, 4150-546 Porto, Portugal; 4 Collective Learning Group, MIT Media Lab, Massachusetts Institute of Technology, Cambridge, MA 02139, USA; 5 INESC-ID and Instituto Superior Técnico, Universidade de Lisboa, IST-Taguspark, 2744-016 Porto Salvo, Portugal; 6 ATP-group, 2744-016 Porto Salvo, Portugal; 7 Centro de Biologia Molecular e Ambiental, Universidade do Minho, 4710-057 Braga, Portugal; 8 Departamento de Matemática e Aplicações, Universidade do Minho, 4710-057 Braga, Portugal

**Keywords:** complex adaptive networks, early modern history, network analysis, trust, reputation

## Abstract

Little is known about the structural patterns and dynamics of the first global trading market (FGTM), which emerged during the sixteenth century as a result of the Iberian expansion, let alone how it compares to today's global financial markets. Here we build a representative network of the FGTM using information contained in 8725 (handwritten) Bills of Exchange from that time—which were (human) interpreted and digitalized into an online database. We show that the resulting temporal network exhibits a hierarchical, highly clustered and disassortative structure, with a power-law dependence on the connectivity that remains remarkably robust throughout the entire period investigated. Temporal analysis shows that, despite major turnovers in the number and nature of the links—suggesting fast adaptation in response to the geopolitical and financial turmoil experienced at the time—the overall characteristics of the FGTM remain robust and virtually unchanged. The methodology developed here demonstrates the possibility of building and analysing complex trading and finance networks originating from pre-statistical eras, enabling us to highlight the striking similarities between the structural patterns of financial networks separated by centuries in time.

## 1. Introduction

The navigating routes established by the Portuguese and Spaniards during the sixteenth century linked all continents [1–12]. Lisbon and Seville became ports of entry in Europe and trading hubs of global goods, and the world witnessed the emergence of the first global trading market (FGTM) [4,5,13,14], which required a common financial mechanism to support a trading system of increasing complexity. Merchants employed Bills of Exchange (BoEs) as the most common payment instruments for transactions and also as an efficient tool of financial credit, allowing merchants to execute commercial exchanges in situations in which they were no longer able to receive their payment directly from the hands of the buyer. Bankers, too, also employed BoEs to enable money transfers capable of sustaining long-distance transactions. International fairs became, at that time, privileged *fora* in which to set accounts and balances between merchants and bankers. As a result, BoEs provide crucial information regarding the paths and characteristic times that accrue to the money transfers associated with most financial transactions. Similar to the global financial market (GFM) of today, following the money transfers in the sixteenth century allows us to characterize the FGTM, the overall structure of which remains essentially unknown to date.

To this end, we used information contained in an online database recently created [15] accumulating records pertaining to approximately 21 000 BoEs of a representative merchant and banker of that time, the Castilian Simon Ruiz (SR) [16,17]*.* By all measures, this is a rare dataset, given the obvious limitations of dealing with historical data from this pre-statistical period. Despite the fact that the FGTM was here built upon BoEs of a single merchant (not many such data sources survived to date), the resulting network spans the entire world, as SR maintained contacts with merchants settled both inside and outside Europe. It is also noteworthy that the trading partners of SR include a significant part of the main trading merchants and bankers that were active during this period of early modern history, such as the Fugger, the Grimaldi or the Spínola, and other powerful Genoese merchant–bankers [18]. In Europe, partners spread from the entire Iberian Peninsula to German cities such as Augsburg and Hamburg, from Antwerp or London to Genoa or Rome. But there are partners also in Spanish America, Brazil, Cape Vert and India [19]. Out of these documents, about 9000 BoEs contain information regarding money transactions with clearly recognizable roles and identifications, suitable to be used in the present study. In the electronic supplementary material, figure S2 and table S1, we provide more detailed geographical information regarding the European nodes that incorporate the network built from the online database. Moreover, the dataset spans half a century of transactions, allowing for a temporal analysis of the FGTM, here represented by a time-varying complex network [20–23] of financial parties.

**Figure 1.**
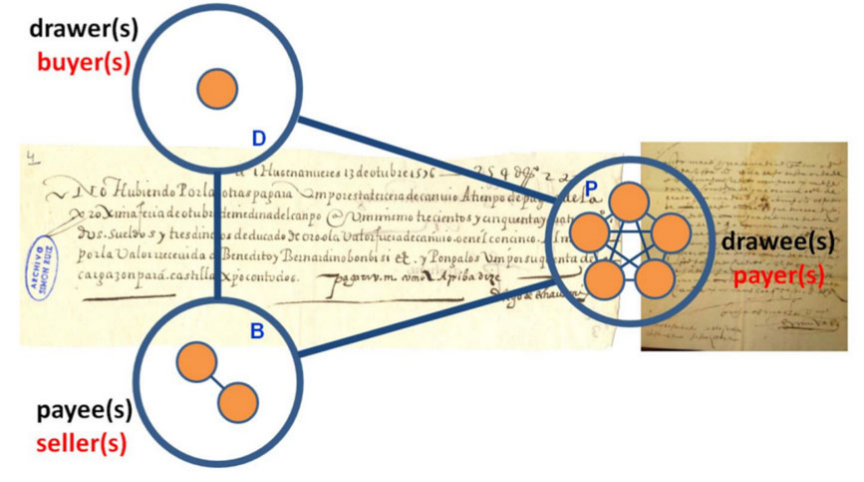
Bills of Exchange (BoEs). Structure and money circuit accruing to each BoE, involving typically three main roles: drawer (D) who, for example, wants to pay for a product or wants to transfer a certain amount of money to the payee (the beneficiary or seller (B), located at a distant place and using, possibly, a different currency) and the drawee or payer (P), who secures payment to B. The background images correspond to scans of typical BoEs extracted from the archive of SR.

From a network perspective, each BoE leads to the appearance of at least one triangular motif in the network, linking the drawer(s), payee(s) and drawee(s)—associated with each transaction, as illustrated in figure 1. However, often the number of individuals designated in one BoE is more than three—more precisely, an average of five (see the electronic supplementary material). In such a case, our network transcription of the data assumes that, in each BoE, all individuals performing the same role form a clique, as illustrated in figure 1 (in the electronic supplementary material, we provide further details of the network construction also exploring other motif templates in building the network, which support the robustness of the results discussed in the following).

## 2. Material and methods

The network was built from data present in 8725 handwritten BoEs [15] (covering the period 1550–1606) that are part of the SR archive. Although the SR company persisted until 1606, under the hands of SR's heir, his nephew Cosme Ruiz, we used only 7908 BoEs, corresponding to the BoEs issued before SR's death in 1597. BoEs were human interpreted and their content inserted in a computer database [15], which was used to build the networks discussed here. In the electronic supplementary material, we provide details on this dataset [15] and the procedures used to build the network providing representative information of the FGTM.

The data consist of a list of individuals and the roles assigned to them in each BoE. Using this information, the links between individuals were established according to the scheme depicted in figure 1. Because of the triangular motif of figure 1, each letter leads to a clique between all participants, and we assumed that links were undirected and unweighted, and ignored self-interactions. Other motif templates explored in the electronic supplementary material, and which do not rely on a triangular relation between three main roles, do not necessarily generate a clique for each letter. In all cases, the FGTM results from the accumulation of all structures associated with the information contained in each BoE. This led to a global network (encompassing the entire SR period of activity) made of 4992 distinct individuals and 30 654 links with a diameter of five links (table 1). Several observables were computed from the networks (table 1), in particular the degree distribution (*D*(*k*)), average path length (APL), degree assortativity (*k*_nn_(*k*)) and clustering coefficient (CC), following the usual definitions [24,25]. Hierarchical analysis of each network was done studying the distribution of the average CC per degree [26].

**Table 1.** Characterization of the FGTM for the merchant and merchant-and-banker periods. (For the two periods identified, we provide data for: the number of nodes (*Z*), the number of links (*L*), the maximum degree (*k*_max_), the network diameter (*d*), the average path length (APL), the average clustering coefficient (CC) as well as the scaling associated with the cumulative degree distribution (*C*_D_(*k*)), degree distribution (*D*(*k*)), average clustering coefficient of nodes with degree *k* (*C*(*k*)) and the average degree of neighbours of nodes with degree *k* (*k*_nn_(*k*)). See Material and methods and the electronic supplementary material for details.)

	merchant	merchant-and-banker
node number (*Z*)	711	4281
link number (*L*)	3293	27 361
average degree 〈 *k* 〉)	9.26	12.78
maximum degree (*k*_max_)	562	3165
diameter (*d*)	4	5
average path length (APL)	2.32	2.41
clustering coefficient (CC)	0.76	0.78
*C*_D_(*k*)	∼*k*^−1.38^	∼*k*^−1.10^
*D*(*k*)	∼*k*^−2.38^	∼*k*^−2.10^
*C*(*k*)	∼*k*^−0.78^	∼*k*^−0.77^
*k*_nn_(*k*)	∼*k*^−0.6^	∼*k*^−0.59^

## 3. Results and discussion

**Figure 2.**
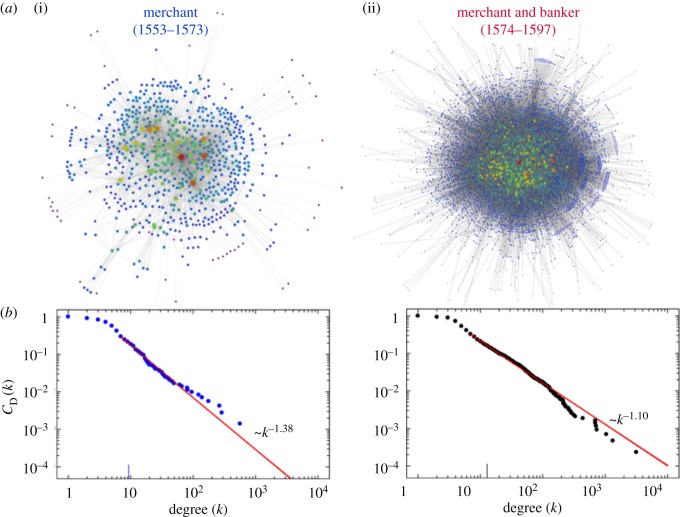
Trade networks of the sixteenth century. (*a*) Both panels represent networks built from the information gathered in [27], reflecting the two distinct periods of activity of SR identified ((i) merchant period; (ii) merchant-and-banker period). Nodes represent individuals who participated in the trade, whereas links connect trading individuals. Nodes are drawn with a temperature colour gradient reflecting their connectivity; thus, hubs are the warmest nodes (red). (*b*) Cumulative degree distributions of the networks are represented on the top (blue dots). The straight red lines provide a power-law fit following the procedure proposed in [28]. This methodology assumes the existence of a lower bound (*k*_min_) and takes into account a goodness of test fit as a means to evaluate the quality of the fit. The exponents of the power-law scaling are indicated.

Being a relevant player of the FGTM, SR was active for an unusually long period of over 40 years (1553–1597) during which he traded not only with all the other key players of the FGTM [1,4,5,13,14] but also with many others. He started as a merchant (1553–1573), subsequently expanding into banking activities (1574–1597). This new business avenue translated into a significant increase of his presence in the FGTM network, as illustrated in figure 2. Indeed, adding banking to his merchant activities resulted in sizeable changes in several features of the network, such as a sixfold increase in the network size and an eightfold increase in the number of partnerships, among many others listed in table 1.

Furthermore, the fact that SR conducted his activity in a period of great turmoil in Europe means that the types of commodities traded by SR were very different. While he started his business affairs as an importer of French textiles into the Peninsula market (possible because of the Treaty of Cateau-Cambrésis), he subsequently invested in American Indies commodities (until the crisis in Seville during the 1560s) and informally in spices and slaves jointly with his Portuguese partners [18]. As an example of a successful merchant of his time, Ruiz was non-specialized, investing in several profitable commodities, and choosing his partners depending on each different kind of business and on each production and consumption markets [29].

After 1574, SR tried to offer the Castilian Crown his accumulated capital to invest in *asientos* to support the Castilian wars with the Protestant Low Countries, although the first contract with the *Consejo de Hacienda* was only signed in 1576 [30]. Until the end of the 1570s, he invested a huge amount of money, especially in 1577 and 1578, when SR moved his investments from the so-called *asientos* of Flanders to the *asientos* of Madrid, investing higher amounts, but obtaining greater profits, namely obtaining silver *licencias de saca* outside Spain. When after 1578–1580, the Genoese recovered their role as the main financiers of the Spanish Crown, SR realized that he could not compete with them and joined them in their consortium until the end of the 1580s, especially owing to the decline of Lyon as a financial centre, where his traditional major partners were struggling to survive the Wars of Religion in France.

SR also invested in lending to some private persons, such as some of the *Grandes* of Spain, among them the Duke of Bejar, the Count of Portalegre, the Count of Nájera or the Duke of Aveiro. He also transferred money between religious institutions. After the mid-1580s, SR invested in a new financial opportunity: the curial market in Rome, i.e. the market of ecclesiastical licences and benefits, for which the Iberian Peninsula had the largest demand in all Christianity [31]. However, he held on to his trading activity but now focused on first-need products. Via France, SR imported Northern European wheat to support bad harvest years in Portugal and Spain (direct trade with The Netherlands was prohibited during the Eighty Years' War), and salt from Brittany and Normandy to Castile [18,32–35]. The diversity of business opportunities in which SR was involved implied different business partnerships and the multiplication of nodes in the network of the merchant-and-banker period. It was also a means of splitting risk in financial business, because his liquidity would be less exposed to a partner's or debtor's default. This variety is also reflected in the geography and nationality of the agents involved in each period (electronic supplementary material, figure S2).

By keeping track of the first and last date at which BoEs were established by the same two nodes, our analysis leads to an exponential distribution of link duration, with a characteristic lifetime for business associations of approximately 9 years. Moreover, links that last longer are those that connect those individuals with a higher number of partnerships in the financial network (see the electronic supplementary material, figure S5)—that correspond to those depicted in figure 2 with warmest colours (following a colour gradient which is a function of connectivity), located preferentially at the centre of the network.

Given that *trust* was found [18,36–38] to be one of the main characteristics positively correlated with link creation in trading networks of the early modern age, contrary to kinship, religion and social status, this also suggests that the hubs of this network were occupied by those individuals with the highest reputation for being trustworthy.

As figure 2 illustrates, the cumulative degree distributions of both networks, where degree here represents the number of partners (links attached) that each individual (node) has (that is, engages in businesses with), exhibit a broad-scale or scale-free (power-law) behaviour [39], the associated degree distributions exhibiting exponents of 2.38 for the merchant period and of 2.10 for the merchant-and-banker period. Further analysis of both networks leads to the results provided in table 1. Altogether, these results show that the FGTM exhibits a scale-free distribution with a high clustering coefficient, being, however, disassortative in degree—i.e. there is a preference for nodes to attach to others that differ in the number of partners. Moreover, the power-law scaling of the degree-dependent clustering coefficient (*C*(*k*) in table 1) suggests that both networks exhibit a hierarchical nature [26], possibly associated with the process of their formation.

In the electronic supplementary material, we show that these features remain robust to changes of the template motif used in building the FGTM. The variant of the FGTM obtained by merely following money transfers (see the electronic supplementary material) exhibits the same fat-tailed distributions with the same exponents already provided in table 1.

All macroscopic features of the FGTM are remarkably similar to what is known for present-day interbank transactions around the globe [40–45]. It is important to note that there is no information available enabling a full mapping of today's GFM, yet several representative networks are available from the mapping of interbank transactions within different countries [40–45] (e.g. Austria, Brazil, Russia, UK and USA). Both data are comparable because these present studies refer to financial flows which, similar to early modern BoEs, are actually not real and not physical. BoEs in the FGTM were such a mechanism of interbank (national or international) remittance that did not involve the movement of physical funds [46,47].

Interestingly, all these contemporary networks exhibit features very similar to each other and, importantly, also with the representative sample of the FGTM that we built here based on the SR records. In particular, and similar to the FGTM, they portray a power-law degree distribution and a power-law dependence of the clustering coefficient, the latter suggesting a hierarchical nature [26]. This being the case, different branches of the full network (either the FGTM or the GFM) are expected to exhibit properties self-similar to those of the full network. Furthermore, both the FGTM and the GFM are found to be disassortative, with the exponents of the power-law degree distributions being approximately 2.10 for the merchant-and-banker period of the FGTM and very similar for the GFM [40–45] (with values ranging 2.0–2.3, whenever comparable data are reported). The similarity found between the features of the FGTM and those of the present GFM is quite surprising, given the disparate means available for doing business. Indeed, while at present the Internet allows for very fast and efficient money transactions that take a fraction of a microsecond, in the sixteenth century, correspondence (and personal travelling to international fairs, whenever possible) were the fastest means to exchange non-local information (and a precious means of gathering information, at present, from that time). Nonetheless, and in spite of the paradigm shifts that separate the infrastructure supporting money transfers between the two periods, what we obtain is that the structural patterns of the network of the old and new financial markets did not change macroscopically. Needless to say, these similarities neither imply nor refute the possibility that the underlying mechanisms of establishing trade in the sixteenth and twenty-first centuries are the same.

**Figure 3.**
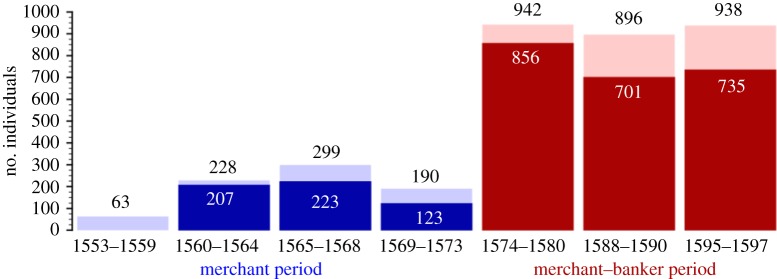
Network size per historical period. Values on top of each bar show the number of individuals participating in BoEs in each period indicated. The numbers inside each bar indicate the number of new individuals appearing in each period, compared to the previous one, and define the height of the dark blue (red) bars. In all cases, most of the individuals appearing in each period were not present in the previous period, reflecting the dynamic nature and adaptability of the FGTM network.

During the 40 years of activity of SR, Europe witnessed four major country bankruptcies and was immersed for most of the time in wars that severely delayed the occurrence of international fairs [48] (see the electronic supplementary material, figure S1). Despite the fact that the trading partnerships of SR encompassed different regions in the globe with diverse currency systems and different financial mechanisms, global trade depended upon the flow of silver, which was in the hands of European merchants [49,50]. Consequently, all the credit market was based in Europe and its exchange fairs. Thus, how was it possible for SR to maintain a successful business activity in the midst of so many financially disruptive events? To answer this question, we use at profit existing historical analysis [18], where the seven most significant periods of the business activity of SR were identified, and use network analysis to perform a comparative study of these seven periods. Results are shown in figure 3, where we distinguish those trading partners of SR that remain in the network across contiguous periods from those that enter the network in the new period. The homologous variations between periods *P*_previous_ and *P*_actual_ shown in figure 3 are computed by finding the number of SR first neighbours during *P*_actual_ who were not neighbours of SR during *P*_previous_. This number is subsequently normalized via division by the total number of first neighbours during *P*_actual_.

The results in figure 3 portray a remarkable flexibility and adaptive capacity in establishing new links in different periods, a feature that we have already alluded to before when reporting historiographical evidence for the banking period of SR. Indeed, figure 3 shows that most of SR's partners in period *n* were no longer active in period *n* + 1, pointing to a very dynamic network in which links are rapidly created and others destroyed.

Interestingly, in each period, the overall network properties do not change appreciably (electronic supplementary material), despite the fact that such a dynamic network has a direct impact on the nature of the goods traded and money transfer circuits explored, possibly reflecting a rapidly changing geopolitical scenario. These network features also find echo in historiographical records: the turbulent events occurring in the second half of the sixteenth century provided significant differences between the two periods, as shown in table 1 and figure 3. It was the Crown's suspension of payments to the Royal Treasure's creditors in 1575, which allowed the entry of Spanish merchants into the business of public debt, which in turn enabled them to accumulate the amount of capital needed to invest in private banking [17,30]. That could also be seen as a means of escaping the decadence of the Medina del Campo fairs [18,48,51]. Both reasons led to a change of the headquarters of SR's company—from Medina to Madrid (see the electronic supplementary material, figure S2).

In fact, the historical conjuncture had so much impact upon the number of nodes and links involved in the structures of BoEs that in the Spanish Treasure bankruptcy of 1596 and in the years before and after, the number of individuals and the number of BoEs decreased (electronic supplementary material, figure S3), affecting the preference of partnerships with some business groups. The preference for Portuguese traders was because of their relative immunity from the financial disaster of the Piacenza fairs (led by the Genoese) and their disposable capital in Antwerp, so that SR continued to participate in *asientos* and sustain the Habsburg conflicts in Europe by investing in public debt [31,35] (see also the electronic supplementary material, figure S1) [30,34]. Also, the anticipation of a decreasing financial activity (decrease in the number of BoEs—electronic supplementary material, figure S3) went along with a strategy of reinforcing businesses with older partners, increasing the degree of trust. Therefore, through different mechanisms of partner choice and different investments, SR was able to adapt to difficult circumstances.

## 4. Conclusion

Although there are no similar topological studies of other early modern financial networks, historians have already underlined the use of similar strategies during the seventeenth and the eighteenth centuries. Even with public banks already functioning as in eighteenth century France, the spreading of investments in different types of business and using different partners in order to reduce risk, as well as the establishment of hierarchical networks in which older partners are more reliable and whose relationships are more stable in time, continued to persist [27,52].

Our results impact different dimensions of our knowledge of the historical origins of economic globalization. On the one hand, we show how a combination of historical analysis and a quantitative analysis of the complexity of historical financial patterns—through modern network science and other methods—may provide a means to circumvent the natural lack of consistent data, enabling also a comparison across distinct periods. On the other hand, our comparison between branches of the sixteenth century FGTM and branches of the present-day GFM led to striking structural similarities at the macro-scale. Historically, the fundamentally disruptive effect that the navigating routes first established by the Portuguese and Spaniards during the sixteenth century had on humanity led to a reconfiguration of the world economy, to the adaptation of existing financial and information circulation mechanisms and to the establishment of trans-national, trans-religious and trans-cultural networks which allowed the circulation of capital at a global level, such as what we have today [38,53–55]. The existence of a global market economy which shared similar exchange values and credit mechanisms established the foundations, functioning and structure of the first global financial networks. As a result, companies responded to such a global market giving rise to economic patterns that, as shown, are structurally comparable to those of today, even if formally different. In this sense, this work suggests the possibility that financial networks may have similar structures in different chronologies, despite completely different historical contexts.

## Supplementary Material

Supplementary Material to Structural and temporal patterns of the first global trading market

## Data accessibility

The original datasets analysed during the current study are publicly available at http://www.dyncoopnet-pt.org/. All data generated or analysed during this study are included in this manuscript and the electronic supplementary material.
