# Supplementary material for: Structural and temporal patterns of the first global trading market
Source: R Soc Open Sci. 2018 Aug 22;5(8):180577. doi: 10.1098/rsos.180577 (PMC6124087; doi:10.1098/rsos.180577)
Supplement: Supplementary Material to Structural and temporal patterns of the first global trading market [file rsos180577supp1.pdf]

Supplementary Material

Structural and temporal patterns of the first global trading market

Ana Sofia Ribeiro[1,2], Flávio L. Pinheiro[4,5,6], Francisco C. Santos[5,6], Amélia Polónia[2,3], and Jorge M. Pacheco[7,8,6]

[1]CIDEHUS, Universidade de Évora, Apartado 94, 7000-809 Évora, Portugal.

[2]CITCEM, Faculdade de Letras, and [3]Departmento de História e Estudos Políticos Internacionais, Universidade do Porto, 4150-546 Porto, Portugal

[4]Collective Learning Group, The MIT Media Lab, Massachusetts Institute of Technology, MA 02139, USA

[5]INESC-ID and Instituto Superior Técnico, Universidade de Lisboa, IST-Taguspark, Porto Salvo, Portugal

[6]ATP-group, P-2744-016 Porto Salvo, Portugal

[7]Centro de Biologia Molecular e Ambiental, and [8]Departamento de Matemática e Aplicações, Universidade do Minho, 4710-057 Braga, Portugal

11 pages including 7 Figures and 2 Tables

1. INTRODUCTION

Information on the First Global Trading Market (**FGTM**) is scarce. One of the best documented merchants of this period of time is the Castilian *Simon Ruiz* (**SR**), who typifies the portrait of the successful Merchant–Banker from the 16th century[1]. The archive of his trading and finance company is among the few which remained to date. Most of **SR** activities took place during the second half of the 16th century (1553 - 1597), where social and financial turbulence were the rule, as illustrated in the "cartoon" summary timeline depicted in Figure S1. During this period, Philip II ruled about 13% of the world territory, adding to it, after 1580, Portugal (and its overseas empire) – resulting in, roughly, 25% of the world territory, spanning 5 continents.

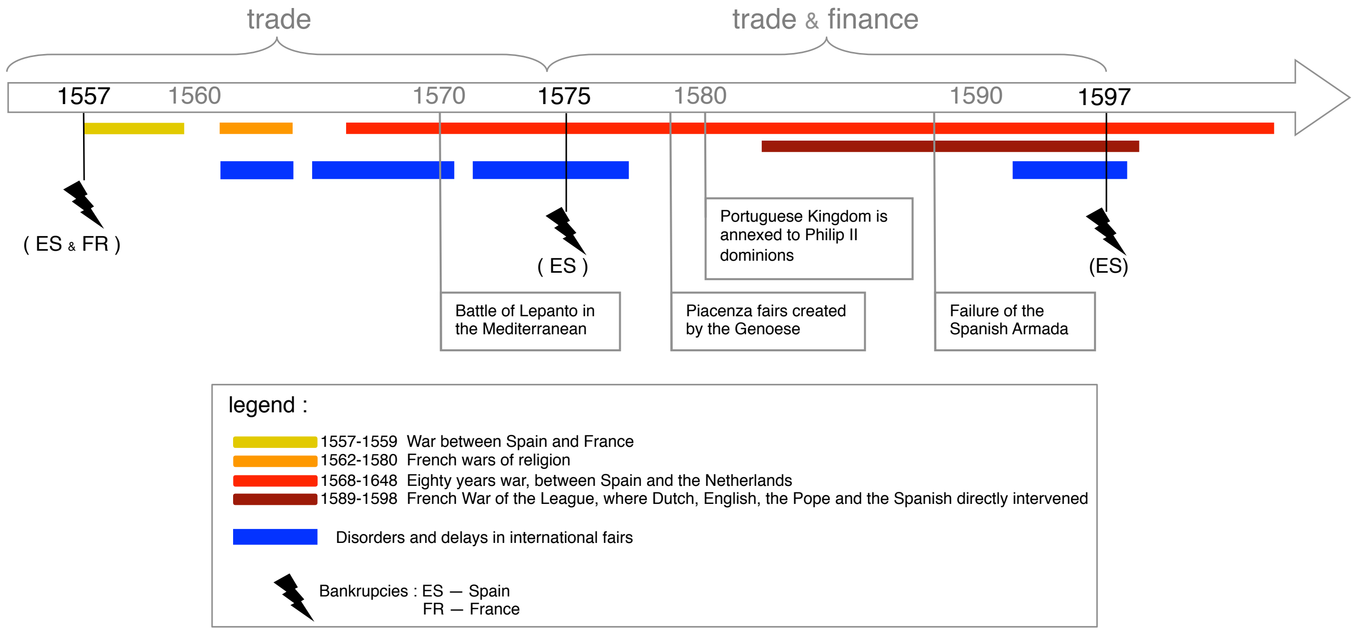

Figure S1. Europe in the second half of the 16th century (1557-1597). An illustrated timeline of Europe during the period of business activity of Simon Ruiz, in which the most relevant events which influenced, directly or indirectly, the business activity of Simon Ruiz, are listed, giving emphasis to wars, country defaults and disorders/delays in financial fairs throughout Europe.

To support and defend such a vast empire, a great financial effort was necessary, and the King resorted to Bankers such as **SR** to complement the (insufficient) internal revenues. Figure S1 emphasizes the political, social and economic turmoil which took place during the business period of **SR** activities, originating, among countless other events, three different sovereign

defaults or bankruptcies (1557, 1575 and 1596) and constant delays of financial fairs, thus compromising the flow of money, given the lack of alternative forms of money circulation[2, 3]. As discussed in the main text, despite such troubled times, **SR** was able to protect and sustain the success of his business (for further details see Ref. [4]). In this supplementary information, we provide additional details regarding both the dataset and methodology adopted in extracting a network from the data.

2. NETWORK CONSTRUCTION AND DATASET CHARACTERIZATION

Bills of Exchange **(BoE)** created a money circuit interconnecting business agents located in different places, performing three standard roles (though roles could be performed by more than one single business agent, see Figure 1 in main text): *i)* The Payee or Beneficiary/Seller (**B**, e.g., the individual selling the goods), the final receiver of the bill and the transacted money; *ii)* The Drawer or Buyer (**D**, e.g., the individual buying the goods), and the Drawee or Payer (**P**, the one who advances the cash to **B**) [5]. From a network perspective, each **BoE** leads to the appearance of at least one triangular motif in the network, associated with a given transaction (see Figure 1). Alternative schemes, associated with different motifs, are discussed below (see Figure S7).

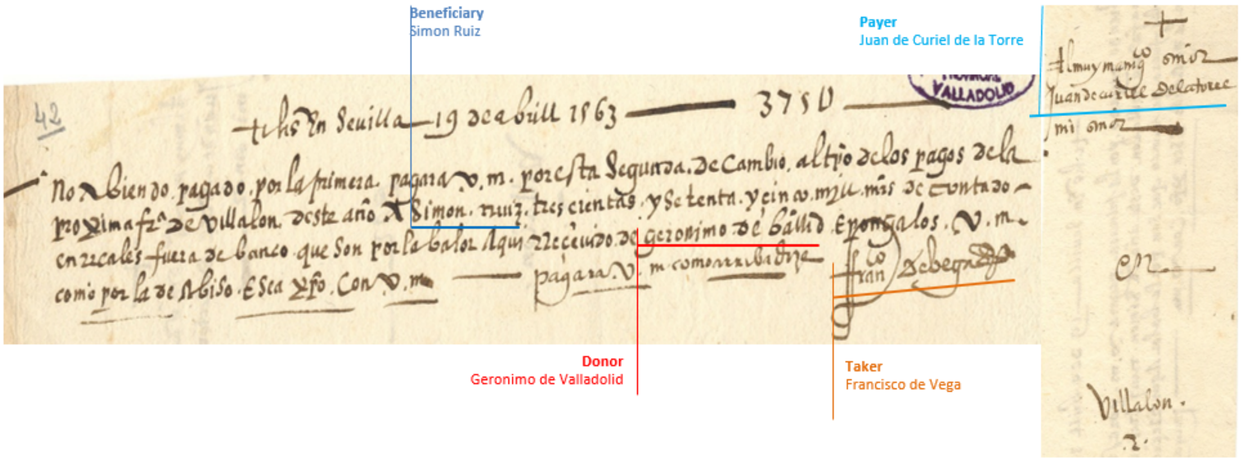

Figure S2. Interpretation and Information extraction from a BoE. In this particular example, all individuals who participate in this **BoE** were identified, together with their role in this **BoE**, as well as the financial relations between the participants. This information is subsequently introduced in a relational database especially built to store all the information contained in the **BoE**.

In order to collect data, paleography must be used to read and interpret every single **BoE**. This way, one identifies not only those that participate in every **BoE** (an explicit example is provided in Figure S2) but also their role, as well as the financial relations between them. This information is subsequently inserted in a relational database especially built to store all this information. This database was developed at Coimbra University by Joaquim Carvalho and his team and is based on micro-historical research with a strong emphasis on network analysis and prosopography [6]. The database, named *Timelink*, allowed a digital reconstitution of each **BoE**, such that financial relations were automatically established between all agents with attributed financial roles. It further reconstructed individual biographies suggested by the register of persons with similar attributes and relations, allowing to understand the presence and role of a certain individual during the period under investigation [7].

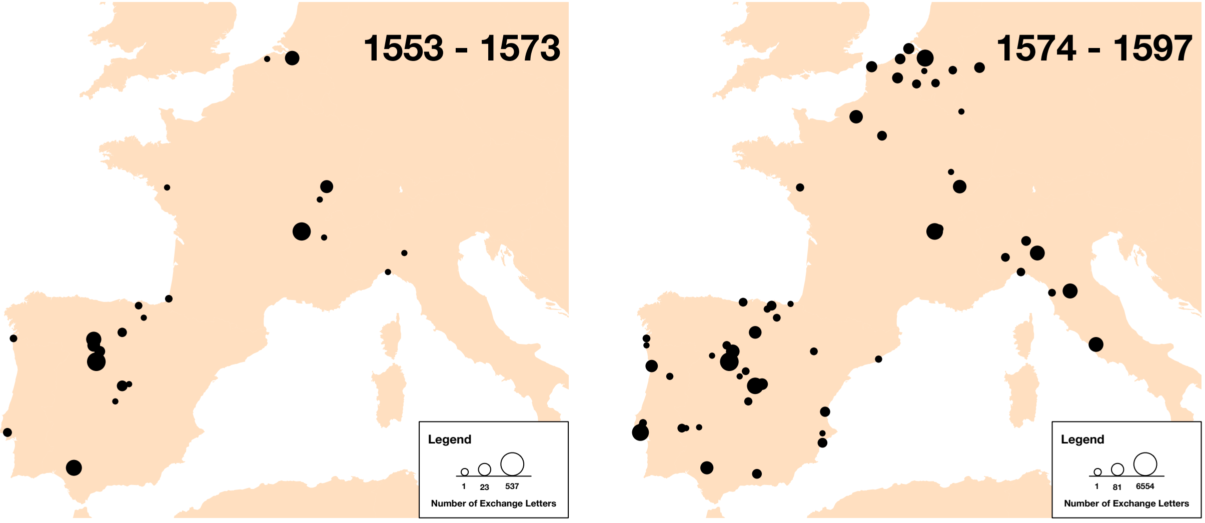

Figure S3. Geographical distribution of the European nodes that incorporate the network built from the database, for each period indicated. Each dot identifies the geographical location of a **SR** partner, the size being proportional to the number of letters of exchanged associated with that location. As detailed in the main text, the financial activity of **SR**, as well as the complex economic scenario after 1575, led **SR** to diversify his investments and increase the number of partners. In the latter period, one also observes a reinforcement of **SR** partnerships with the Genoese in Piacenza and Antwerp, as well as with Portuguese merchants in the Low Countries and in Rome. Finally, during this period **SR** moved his headquarters from Medina del Campo to Madrid, where he established businesses with the Portuguese in the Atlantic trade and became a creditor of merchants and other social groups all over Spain.

Name	Nr. of BoE	Begin	End
Bernardino Bonvisi	1455	1568	1597
Stefano Bonvisi	981	1568	1597
Antonio Bonvisi	959	1581	1597
Benedito Bonvisi	921	1568	1589
Carlos Veluti	466	1586	1596
Francisco Alonso Maluenda	423	1560	1596
Simao Rodrigues de Evora	401	1577	1597
Heirs of Ludovico and Benedeto Bonvisi	392	1565	1578
Lope de Arziniega	392	1569	1597
Andre Ruiz	384	1553	1579
Pedro de Maluenda	375	1573	1596
Manuel Henriques	346	1575	1596
Christoval de Aldan	314	1595	1596
Nicolau Rodrigues de Evora	307	1575	1596
Baltasar Suarez	290	1579	1590
Joao Pasqual	266	1582	1596
Diego Pardo	260	1576	1596
Antonio Suarez de Vitoria e companhia	260	1579	1596
Diego de la Peña	252	1577	1596
Juan Ortega dela Torre	237	1568	1596
Christoval Balbani	224	1582	1596
Francisco de Bobadilla	221	1585	1597
Andres Ximenes	214	1577	1596
Fernam Ximenes	213	1574	1597
Sebastian Pasqual	213	1579	1597
Jeronimo de Noronha	205	1585	1597
Martim Perez de Varron	204	1574	1597
Manuel da Veiga	196	1588	1597
Lopo Rodrigues de Evora	194	1575	1597

Table S1. The 30 longest-lasting partners of **SR** are identified for the banker-and-merchant period (post 1574), together with the period during which **SR** maintained businesses with each of them. The table also reveals the diversity of partners involved: From the Portuguese Ximenes and Rodrigues de Évora to the Tuscans Balbani and Bonvisi, **SR** opted for those who were best located and ready to invest, namely in the financial activity.

SR Company archive comprises more than 21000 **BoE** [8], thus providing longitudinal information concerning the contact structure of **SR**. These contacts include most of the main trading Merchants and Bankers that were active during this period of Early-Modern History, also reflected in the geography and nationality of the nodes involved (see Figure S3 and Table S1). Our data, however, does not contain all information in the database, as *i*) some of it did not contain all the information required to become valid data regarding the present network analysis

and *ii*) we concentrated on those **BoE** related to financial activities. As a result, the data comprises 8725 **BoE** of which 7908 were issued previous to Simon Ruiz death in 1597.

Figure S4 shows the volume of activity registered through the number of **BoE** issued (left panel) and the number of different participants (right panel) per year. These results show that the change in the main role of activities discussed in the main text (i.e. from *Merchant* to *Merchant-and-Banker*) is correlated with an increase in both the overall number of different participants and in the number of **BoE** issued (blue dots indicating the *Merchant* while red dots refer to the *Merchant-and-Banker* periods).

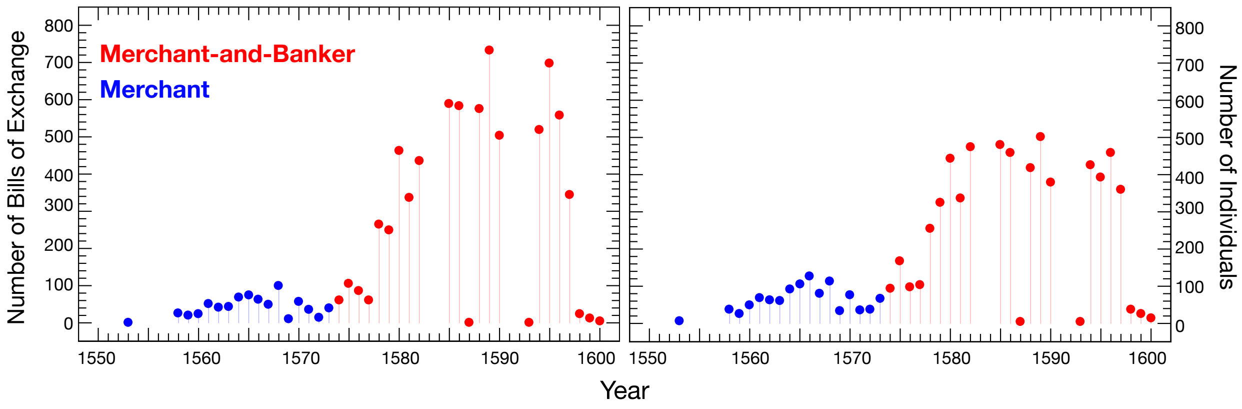

Figure S4. Left: Number of *Bill of Exchange* issued in each year and **Right**: Number of different individuals identified in the *Bills of Exchange* per year. Blue symbols relate to the values obtained during the Merchant Period while Red symbols refer to the Merchant-and-Banker Period. The gray area indicates a region during the Banker Period whose pattern matches the activity during the Merchant Period.

Apart from the larger volume of data and different type of activity, in both periods, the distribution of the number of participants in each **BoE** is characterized by a bell shape curve with expected value close to 5 in both cases, *i.e.* on average each **BoE** had 5 participants, as shown in Figure S5.

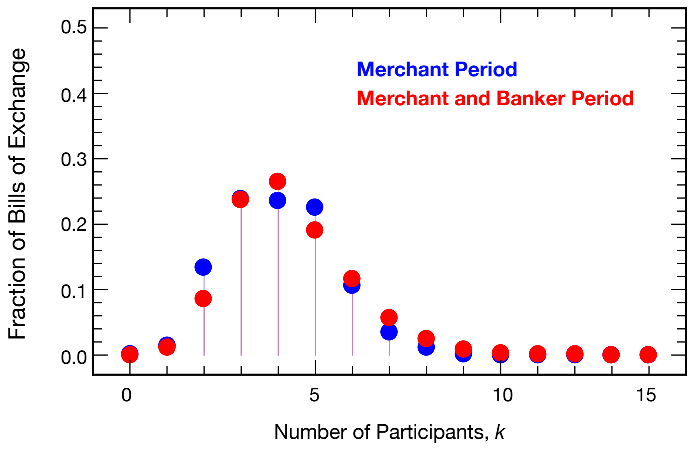

Figure S5. Distribution of the number of participants per *Bill of Exchange* (BoE). Blue (Red) symbols show the fraction of BoE with k participants for the Merchant (Merchant-and-Banker) periods.

A network perspective on this dataset suggests that, despite differences in the overall magnitude of nodes and links, both periods (*Merchant only* and *Merchant-and-Banker*) exhibit networks with similar topological characteristics, which, in turn, show close resemblances with today's financial networks [9-14]. In particular, as illustrated in Figure 2 of the main text, both periods exhibit degree distributions — $D(k)$, defined as the fraction of nodes with degree k — with power-law (fat-tailed) behaviors ($D(k) \sim k^{-\gamma}$). The network preceding 1574 has a degree distribution with an associated exponent $\gamma \approx 2.38 \pm 0.01$ as opposed to $\gamma = 2.10 \pm 0.01$ associated with the network that characterizes **SR** activities afterwards. This difference emerges from the fact that, as a "*Merchant*", **SR** had at most 400 different partners, whereas as a "*Merchant–and–Banker*", **SR** reaches a staggering number of more than 2000 different business partners.

Both periods exhibit large values of the overall *clustering coefficient* (C) (C=0.76 and C=0.78 for the Merchant and Merchant-and-Banker periods respectively), meaning that partners of two connected financial players will tend to be also connected (see Table S2). When such measure is computed as a function of the degree k, we reach a distribution of clustering coefficients that also follows a power-law $C(k) \sim k^{-\beta}$, which can be seen as a fingerprint of hierarchical organization associated with a modular network structure [15]. This suggests that **SR**

traded with very different classes or groups of individuals from different economic communities/interests. Interestingly, these networks also show evidence of degree *disassortativity* [16-18], which indicates that there is an overall tendency for nodes to attach to others with different degree. This feature has been assessed by computing a degree correlation function $k_{nn}(k)$, defined as the average degree of neighbors of nodes with degree k. Since this function follows $k_{nn}(k) \sim k^{\mu}$ with $\mu < 0$, one may say that hubs prefer to link to low-degree nodes (see Table S2) [16, 17]. This feature is also often associated with networks with power-law degree distributions with an exponent $\gamma < 3$ [17], as it is the case here.

Overall, when compared to trading networks of the 21st century [9-14] (here, different branches of the **GFM**), we find remarkable similarities of scaling, clustering and hierarchy. Indeed, present day financial networks also exhibit fat tailed degree distributions, large clustering [19] as well as disasssortativity.

When the time-dependence of the dataset is considered, we observe a large spectrum of short and long-lived partnerships, with an average duration value of 9 years. The duration of each partnership was measured tracing the first and last time that an individual participated in network. Interestingly, those links that last longer are those that link those individuals with higher degree. This is shown in Figure S6, where we plot the relationship between the average degree (full circles) and degree variance (error bars) of individuals in terms of the duration of the partnerships they participate.

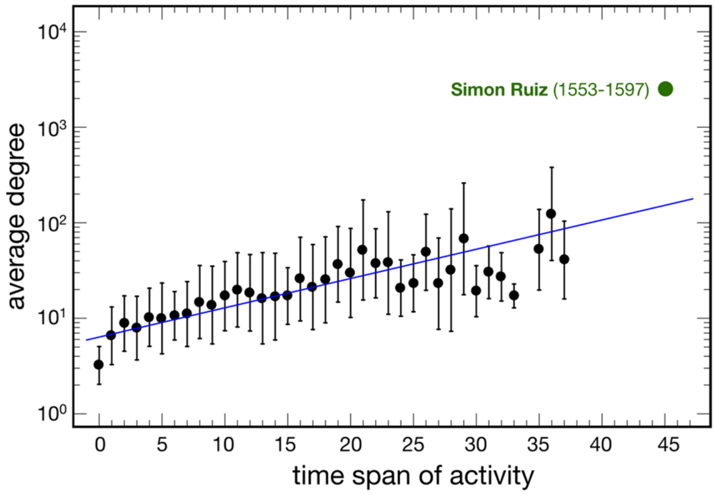

Figure S6. We plot in the Y-axis the average degree (full circles) and degree variance (error bars) of all individuals whose partnership duration is given by the corresponding value in the X-axis. The positive slope of the linear fit indicates that those individuals with a higher average degree are precisely those that remain longest in partnerships.

The transcription of the data contained in the dataset of Ref [8] onto a network, as discussed in the main text, is not unique. However, and apart from obvious quantitative changes, related to a different number of links introduced by each **BoE**, the main qualitative conclusions remain robust when different motifs are adopted when the dataset of **BoE**s is translated into a graph. To illustrate this issue, here we analyze the three different schemes pictured in Figure S7, the last one discussed already in the main text.

Table S2, in turn, compares the main network properties obtained from the different characterization schemes. Properties include the number of nodes (Z) and links (L), the average degree ($<k>$), average path length (APL), clustering coefficient (C), diameter ($diam$), and also distributions in which other macroscopic properties of the network are unveiled, such as degree correlation function ($k_{nn}(k)$) and the distribution of (local) clustering coefficients ($C(k)$) [17, 18]. Overall, all paradigms generate networks characterized by similar *power-law* degree distributions and high clustering, with a *disassortative* and *hierarchical* character, whose similarities with present day interbank activity networks [9, 20] have been addressed in the main text already.

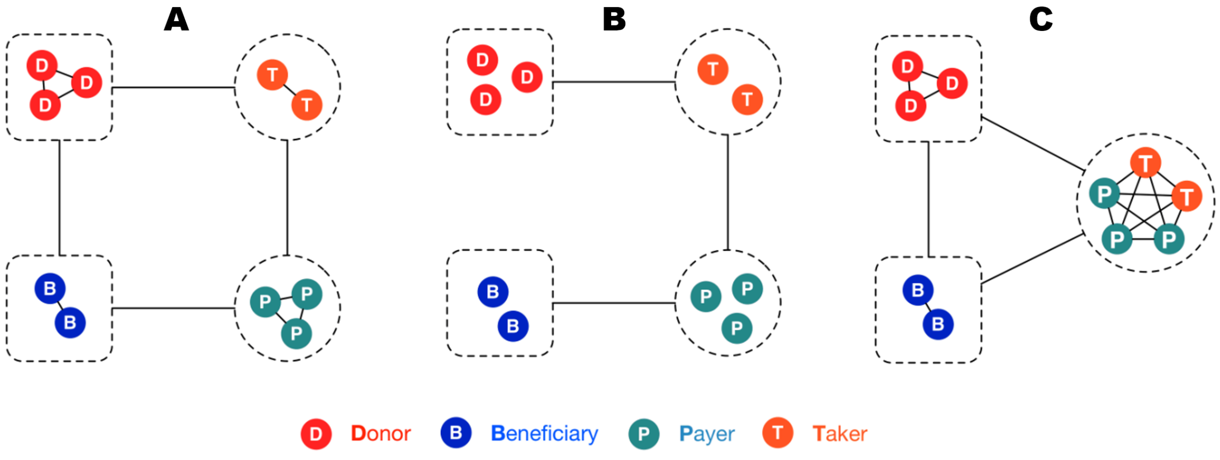

Figure S7 – Prototype schemes proposed for the network of interactions between the participants in a *Bill of Exchange*: Each group consists of individuals performing the same role and is identified by a circle/square with dashed line. Links between groups imply that individuals of one group are connected to all individuals in the other group; links between elements of the same group imply that all individuals of that group are connected in such a way that they form a clique. The scheme **C** corresponds to that adopted in the main text, where Modern Age Takers and Payers were collapsed into a single class. The results in Table S2 below show that this assumption leads to results that reproduce the main features obtained when adopting alternative schemes, more faithful to historical practice, where Takers and Payers were geographically far apart.

	Scheme A		Scheme B		Scheme C	
	**Merchant**	**Banker**	**Merchant**	**Banker**	**Merchant**	**Banker**
Z	711	4281	711	4281	711	4281
L	2544	21803	2032	16885	3293	27361
$<k>$	7.16	10.19	5.72	7.91	9.26	12.78
k_{max}	398	2455	356	1926	562	3165
diam	6	6	7	6	4	5
APL	2.65	2.70	2.83	2.95	2.32	2.41
CC	0.530	0.588	0.29	0.38	0.763	0.785
$D(k)$	$\sim k^{-2.47}$	$\sim k^{-2.18}$	$\sim k^{-2.56}$	$\sim k^{-2.19}$	$\sim k^{-2.38}$	$\sim k^{-2.10}$
$C(k)$	$\sim k^{-0.798}$	$\sim k^{-0.769}$	$\sim k^{-0.74}$	$\sim k^{-0.64}$	$\sim k^{-0.78}$	$\sim k^{-0.77}$
$k_{nn}(k)$	$\sim k^{-0.5}$	$\sim k^{-0.512}$	$\sim k^{-0.54}$	$\sim k^{-0.51}$	$\sim k^{-0.6}$	$\sim k^{-0.59}$

Table S2 Comparison of the main quantities that characterize the graphs for the Merchant and Merchant-and-Banker periods. The quantities tabulated are: the number of nodes (Z); the number of edges (L); the maximum degree (k_{max}), the graph diameter (diam), the average path length (APL), the average clustering coefficient (*CC*), the degree distribution ($D(k)$), the average clustering coefficient of nodes with degree k ($C(k)$) and the average degree of neighbours of nodes with degree k ($k_{nn}(k)$). The results are remarkably robust to different assumptions adopted in setting up the network, namely adopting (or not) a clique linking individuals with the same role in each **BoE** or assuming (or not) 3 roles for each **BoE**.

References

1 Lapeyre, H. 1953 *Simon Ruiz et les asientos de Philippe II*. Paris: SEVPEN.

2 Ruiz Martin, F. 1986 Las ferias de Castilla. In *Historia de Medina del Campo y su tierra*. (ed.^eds. E. Lorenzo Sanz), pp. 269-300. Valladolid: Ayuntamiento de Medina del Campo/ Consejeria de Educacion y Cultura de la Junta de Castilla y Leon/ Diputación Provincial de Valladolid/ Caja de Ahorros Provincial de Valladolid.

3 Yun, B., Comin, F. 2011 Las Crisis de la Deuda Publica en España. *in X Congreso Internacional de la AEHE.*, 2-10 (Seville).

4 Ribeiro, A. S. 2016 *Early Modern Trading Networks in Europe. Cooperation and the case of Simon Ruiz*. Routledge (London, New York).

5 Roover, R. d. 1953 *L'évolution de la lettre de change (XIVe-XVIIIe siècles)*. Paris: Armand Colin.

6 Carvalho, J. M. 2010 Time Link: a evolução de uma base de dados prosopográfica. Coimbra: Faculdade de Letras da Universidade de Coimbra, Portugal.

7 Polónia, A., Ribeiro, A. S., Pinto, S. 2014 Trade networks in the first global age: the case study of Simon Ruiz company: visualization methods and spatial projections. In *Spatial-temporal narratives: historical GIS and the study of global trading networks (1500-1800)*. (eds. A. Crespo Solana), pp. 140-177. Cambridge, UK: Cambridge Scholars.

8 Polónia, A. 2008 DynCoopNet: Portuguese research Project. . *Report to European Science Foundation* , Porto.

9 Soramäki, K., Bech, M. L., Arnold, J., Glass, R. J., Beyeler, W. E. 2007 The topology of interbank payment flows. *Physica A*. **379**, 317-333. (http://dx.doi.org/10.1016/j.physa.2006.11.093)

10 Santos, E. B., Cont, R. 2010 The Brazilian interbank network structure and systemic risk. *Working Papers Series from Central Bank of Brazil, Research Department*.

11 Leonidov, A., Rumyantsev, E. 2012 Russian interbank networks: main characteristics and stability with respect to contagion. *arXiv preprint arXiv:1210.3814*.

12 Afonso, G., Kovner, A., Schoar, A. 2013 Trading partners in the interbank lending market. *Staff Report, Federal Reserve Bank of New York*.

13 Langfield, S., Liu, Z., Ota, T. 2014 Mapping the UK interbank system. *J. Bank. Financ*. **45**, 288-303. (http://dx.doi.org/10.1016/j.jbankfin.2014.03.031)

14 Boss, M., Elsinger, H., Summer, M., Thurner 4, S. 2004 Network topology of the interbank market. *Quantitative Finance*. **4**, 677-684.

15 Ravasz, E., Barabási, A. L. 2003 Hierarchical organization in complex networks. *Phys Rev E*. **67**, 026112.

16 Newman, M. E. J. 2002 Assortative mixing in networks. *Phys Rev Lett*. **89**, 208701.

17 Barabási, A. L. 2016 *Network Science*. Cambridge University Press.

18 Dorogovtsev, S. N. 2010 *Lectures on complex networks*. Oxford: Oxford University Press.

19 Amaral, L. A. N., Scala, A., Barthélemy, M., Stanley, H. E. 2000 Classes of small-world networks. *Proceedings of the National Academy of Sciences*. **97**, 11149.

20 Fricke, D., Finger, K., Lux, T. 2013 On assortative and disassortative mixing in scale-free networks: The case of interbank credit networks. . *Kiel Working Paper*.
